# Association between blood eosinophil count and risk of readmission for patients with asthma: Historical cohort study

**DOI:** 10.1371/journal.pone.0201143

**Published:** 2018-07-25

**Authors:** Marjan Kerkhof, Trung N. Tran, Maarten van den Berge, Guy G. Brusselle, Gokul Gopalan, Rupert C. M. Jones, Janwillem W. H. Kocks, Andrew Menzies-Gow, Javier Nuevo, Ian D. Pavord, Sarang Rastogi, David B. Price

**Affiliations:** 1 Observational & Pragmatic Research Institute Pte Ltd, Singapore, Singapore; 2 AstraZeneca, Gaithersburg, MD, United States of America; 3 University of Groningen, University Medical Center Groningen, Groningen, Netherlands; 4 Ghent University Hospital, Ghent, Belgium; 5 The Peninsula College of Medicine and Dentistry, Plymouth, United Kingdom; 6 Royal Brompton and Harefield NHS Foundation Trust, London, United Kingdom; 7 AstraZeneca, Madrid, Spain; 8 Respiratory Medicine Unit and Oxford Respiratory NIHR BRC, Nuffield Department of Medicine, University of Oxford, Oxford, United Kingdom; 9 Academic Primary Care, University of Aberdeen, Aberdeen, United Kingdom; National and Kapodistrian University of Athens, GREECE

## Abstract

**Background:**

Recent studies have demonstrated an association between high blood eosinophil counts and greater risk of asthma exacerbations. We sought to determine whether patients hospitalized for an asthma exacerbation were at greater risk of readmission if they had a high blood eosinophil count documented before the first hospitalization.

**Methods:**

This historical cohort study drew on 2 years of medical record data (Clinical Practice Research Datalink with Hospital Episode Statistics linkage) of patients (aged ≥5 years) admitted to hospital in England for asthma, with recorded blood eosinophil count within 1 baseline year before admission. We analyzed the association between high blood eosinophil count (≥0.35x10^9^ cells/L) and readmission risk during 1 year of follow-up after hospital discharge, with adjustment for predefined, relevant confounders using forward selection.

**Results:**

We identified 2,613 eligible patients with asthma-related admission, of median age 51 years (interquartile range, 36–69) and 76% women (1,997/2,613). Overall, 835/2,613 (32.0%) had a preadmission high blood eosinophil count. During the follow-up year, 130/2,613 patients (5.0%) were readmitted for asthma, including 55/835 (6.6%) with vs. 75/1,778 (4.2%) without high blood eosinophil count at baseline (adjusted hazard ratio [HR] 1.49; 95% CI 1.04–2.13, p = 0.029). The association was strongest in never-smokers (n = 1,296; HR 2.16, 95% CI 1.27–3.68, p = 0.005) and absent in current smokers (n = 547; HR 1.00, 95% CI 0.49–2.04, p = 0.997).

**Conclusions:**

A high blood eosinophil count in the year before an asthma-related hospitalization is associated with increased risk of readmission within the following year. These findings suggest that patients with asthma and preadmission high blood eosinophil count require careful follow-up, with treatment optimization, after discharge.

## Introduction

Severe asthma exacerbations may result in hospital admissions, relatively rare but important events with adverse implications for patients’ quality of life, health care resource use, and related costs. Approximately 83,000 hospital episodes (including inpatient, day-case, and intensive care episodes) were recorded as related to asthma in England in 2011–2012, representing approximately 3.3 million patients with clinician-reported, diagnosed-and-treated asthma in England during that time [1].

Recent studies have demonstrated an association between high blood eosinophil counts and greater risk of asthma exacerbations, especially in patients with asthma that is not well-controlled [2,3]. Moreover, among patients with severe asthma in a US cohort study, the odds of asthma-related hospital admissions were significantly greater for patients with high blood eosinophil count defined as ≥0.4x10^9^ cells/L than for those with counts of <0.4x10^9^ cells/L [4]. Similarly, in the UK, patients with severe uncontrolled eosinophilic asthma (blood eosinophil count ≥0.3x10^9^ cells/L) experienced over 7 times the number of hospitalizations per year compared with the general asthma population [5], and in Finland, a blood eosinophil count >0.3x10^9^ cells/L was associated with 13% greater rate of hospital admissions (vs. ≤0.3x10^9^ cells/L) among patients with asthma [6]. Targeted therapy for patients with severe eosinophilic asthma can reduce the rate of exacerbations requiring hospitalization and/or an emergency department (ED) visit [7,8].

Patients who are admitted to hospital for asthma-related reasons, such as a severe exacerbation, may be at risk of short-term readmission to hospital. For example, some patients with persistent airways inflammation are at risk of readmission after discharge despite treatment with corticosteroids [9,10]. Predictors of readmission are important to identify as this information could be used to improve in-hospital and post-hospitalization patient management to minimize subsequent readmissions. Several demographic and socioeconomic risk factors for hospital readmissions have been reported for patients with asthma, including older age, greater number of comorbidities, an urban hospital setting, and longer length of hospital stay [11,12]. A recent study found that elevated blood eosinophil count (≥0.3x10^9^ cells/L) in the first blood sample upon hospitalization was associated with a lower incidence of hospital readmissions as compared with an eosinophil count <0.3x10^9^ cells/L [13]. Conversely, for patients with chronic obstructive pulmonary disease (COPD), a recent publication reports an association of increased readmissions with blood eosinophil count ≥0.20x10^9^ cells/L at first hospitalization [14]. The variability in associations may be because blood eosinophils are prognostic and theragnostic.

The aim of this study was to determine if patients hospitalized for an asthma exacerbation were more likely to be readmitted if their preadmission blood eosinophil count was elevated. Our hypothesis was that standard management of asthma exacerbations is insufficient to prevent readmissions for patients who have high blood eosinophil counts in the year preceding a hospitalization.

## Methods

### Data source

We used primary and secondary care data from the Clinical Practice Research Datalink (CPRD) and linked Hospital Episode Statistics (HES) for this historical cohort study of patients with asthma who had been admitted to hospital in England. The CPRD is a large well-validated database, frequently used for medical and health research, that contains de-identified, longitudinal medical records of 5 million patients from >600 UK practices [15]. The linked HES data include detailed information about hospital admissions, ED visits, and outpatient visits to secondary care in England [16]. We used the HES Admitted Patient Care database, which contains records of patients who were admitted to a hospital ward, including patients who visited an ED before admission and those who were admitted to an intensive care unit. Diagnostic and treatment data are recorded in the CPRD using Read codes, while diagnosis data are recorded in HES using International Classification of Disease (ICD)-10 clinical coding and OPCS4 procedural coding.

The study dataset spanned the period from April 1997 through February 2016.

### Study design and patients

Eligible patients were 5 years or older at the time of their most recent asthma diagnosis and had active asthma, which we defined as (1) a diagnostic Read code for asthma qualifying for inclusion in the asthma registry, which general practices in the UK maintain for the Quality Outcomes Framework (QOF) [17], (2) no recorded asthma-resolved Read code after the last asthma diagnosis code, and (3) at least 2 prescriptions for asthma (controller or reliever medication) during 1 baseline year. Patients admitted to hospital with asthma as the primary diagnosis (ICD-10 code J45-J46) were eligible for the study if they had one or more valid blood eosinophil counts recorded during the year before the hospital admission with no prescription for oral corticosteroids within 2 weeks before the eosinophil count.

**Fig 1:**
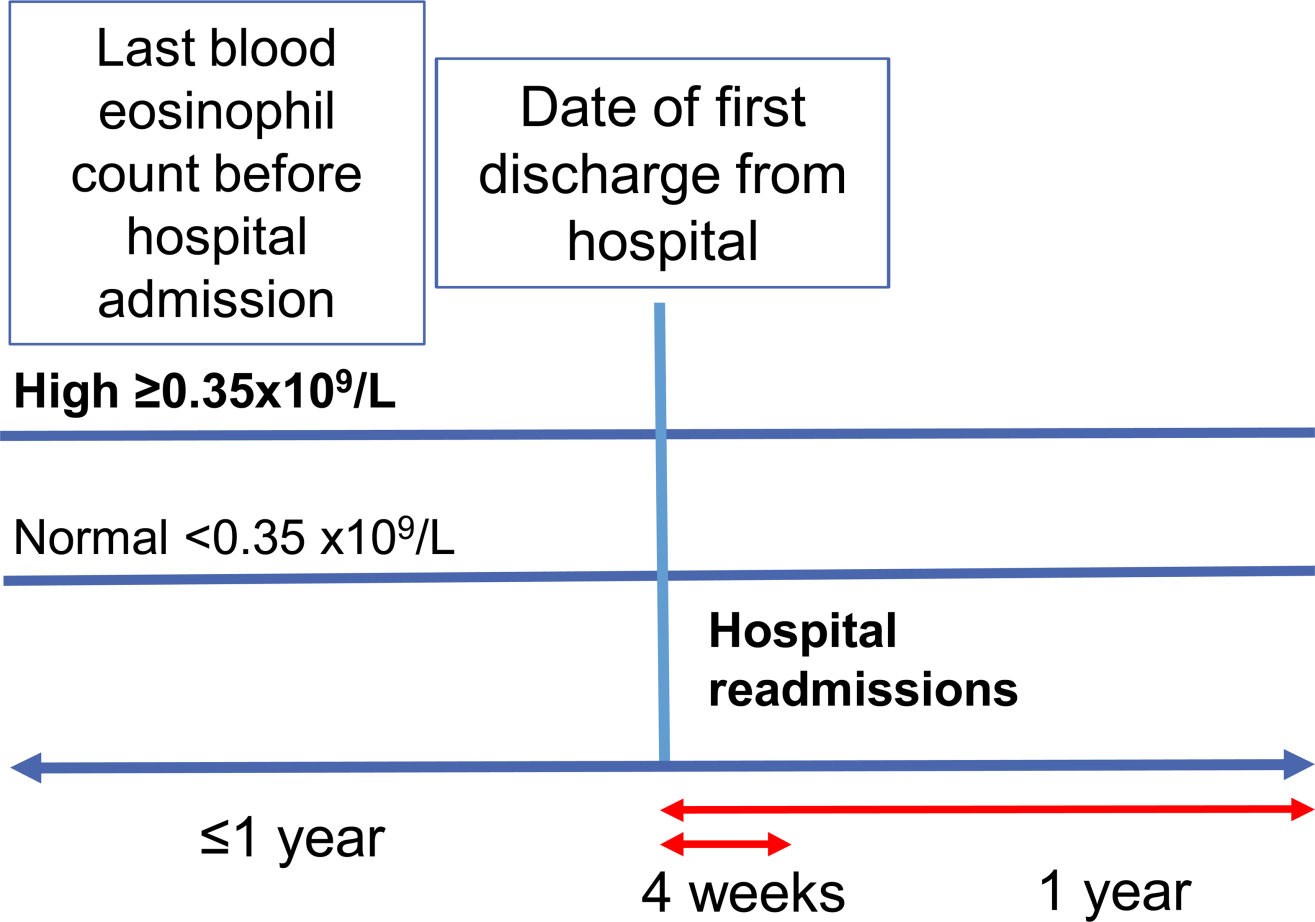
Study design.

Eligible patients had to have available, continuous data throughout the study period (Fig 1), which included ≥1 baseline year before discharge from the hospital for patient characterization and ≥1 outcome year after hospital discharge for follow-up (except for patients who died within 1 year after hospital discharge). We included the first hospitalization recorded for each patient meeting those criteria. A diagnostic Read code for any of the following chronic respiratory conditions recorded at any time was cause for exclusion from the study: bronchiectasis, pulmonary sarcoidosis, hypersensitivity pneumonitis, malignancy of the lungs, interstitial lung disease, and cystic fibrosis. Patients with concomitant diagnosis of COPD were not excluded.

The study was performed in compliance with all applicable local and international laws and regulations and to standards suggested for observational studies, including an independent advisory group, use of an *a priori* analysis plan, and study registration with commitment to publish [18]. The study protocol was approved by the CPRD Independent Scientific Advisory Committee (ISAC approval number 16_236) and registered with the European Union electronic Register of Post-Authorisation Studies (EU PAS Register number EUPAS15869) [19]. No patient identifying information was accessible during the study.

### Outcome assessments

The exposure of interest was the most recent blood eosinophil count measured within 1 year before hospital admission. For patients who had multiple tests in the baseline year, we used the blood eosinophil count (with no oral corticosteroid prescription within 2 weeks prior) that was closest to the admission. A high blood eosinophil count was defined as ≥0.35x10^9^ cells/L (or ≥0.4x10^9^ cells/L when counts were recorded to only 1 decimal place). This value was chosen based on our findings in a prior study in which patients with blood eosinophil counts >0.3x10^9^ cells/L experienced more severe exacerbations and poorer asthma control [3].

The primary outcome was readmission to hospital with asthma as primary diagnosis (ICD-10 code J45/J46) over a 4-week outcome period and over a 1-year outcome period after discharge from the hospital (Fig 1). The secondary outcome was readmission to hospital with asthma as a secondary/subsidiary diagnosis and a respiratory condition as primary diagnosis (ICD-10 codes J00-J99), again observed over 4 weeks and 1 year.

### Statistical analysis

Patients’ baseline characteristics and hospital readmissions were compared between patients with high and normal blood eosinophil counts using Pearson's χ^2^ test of independent categories for categorical variables, and the Mann-Whitney test for continuous variables.

Kaplan-Meier curves were constructed for patients with and without high blood eosinophil count for the maximum follow-up period of 1 year after hospital discharge. Comparisons were made with log-rank analyses, and patients were censored if they died.

Cox proportional hazard regression, with the time from hospital discharge date to the first readmission date as the “survival” time, was performed to estimate hazard ratios (HRs) with 95% confidence intervals (CIs) for the association between high blood eosinophil count and time to readmission, adjusted for potential confounders. The following variables were evaluated for their potential confounding effect on the effect estimate: sex, age, body mass index (BMI), smoking habits, timing of blood eosinophil count relative to the first hospitalization, Charlson comorbidity index (categorical as 0, 1−4, ≥5), comorbidities, and Global Initiative for Asthma [20] (GINA) treatment step (S1 Table). The likelihood of a blood eosinophil count being recorded was greater at dates closer to the hospital admission, and we included the time between recorded eosinophil count and first hospitalization as a confounder in the Cox regression model. Final models were arrived at following a forward-selection procedure, in which variables were added one-by-one and retained if the coefficient for the effect estimate (high eosinophil count) changed by ≥5%. Co-linearity was checked by evaluating variance inflation factors, which were all under 5%. The validity of the proportional hazards assumption was checked by examination of survival curves, and p-values were calculated using a Wald test.

Potential effect modification of smoking status was tested for significance by including an interaction term into the full model. We conducted several sensitivity analyses, repeating the outcome analyses using alternative definitions of high blood eosinophil counts (≥0.25x10^9^ cells/L or ≥0.3x10^9^ cells/L if rounded, and ≥0.45x10^9^ cells/L or ≥0.5x10^9^ cells/L if rounded) and examining outcomes in two subsets of patients: (1) after exclusion of those who initiated inhaled corticosteroids (ICS) after their first asthma-related hospital admission and (2) after exclusion of patients with a concomitant diagnosis of COPD.

Statistical analyses were conducted using IBM SPSS Statistics version 23 (IBM SPSS Statistics, Feltham, Middlesex, UK) and R version 3.0.2 (The R Project for Statistical Computing; https://www.r-project.org/). A statistically significant result was defined as p≤0.05.

## Results

### Patients

**Fig 2:**
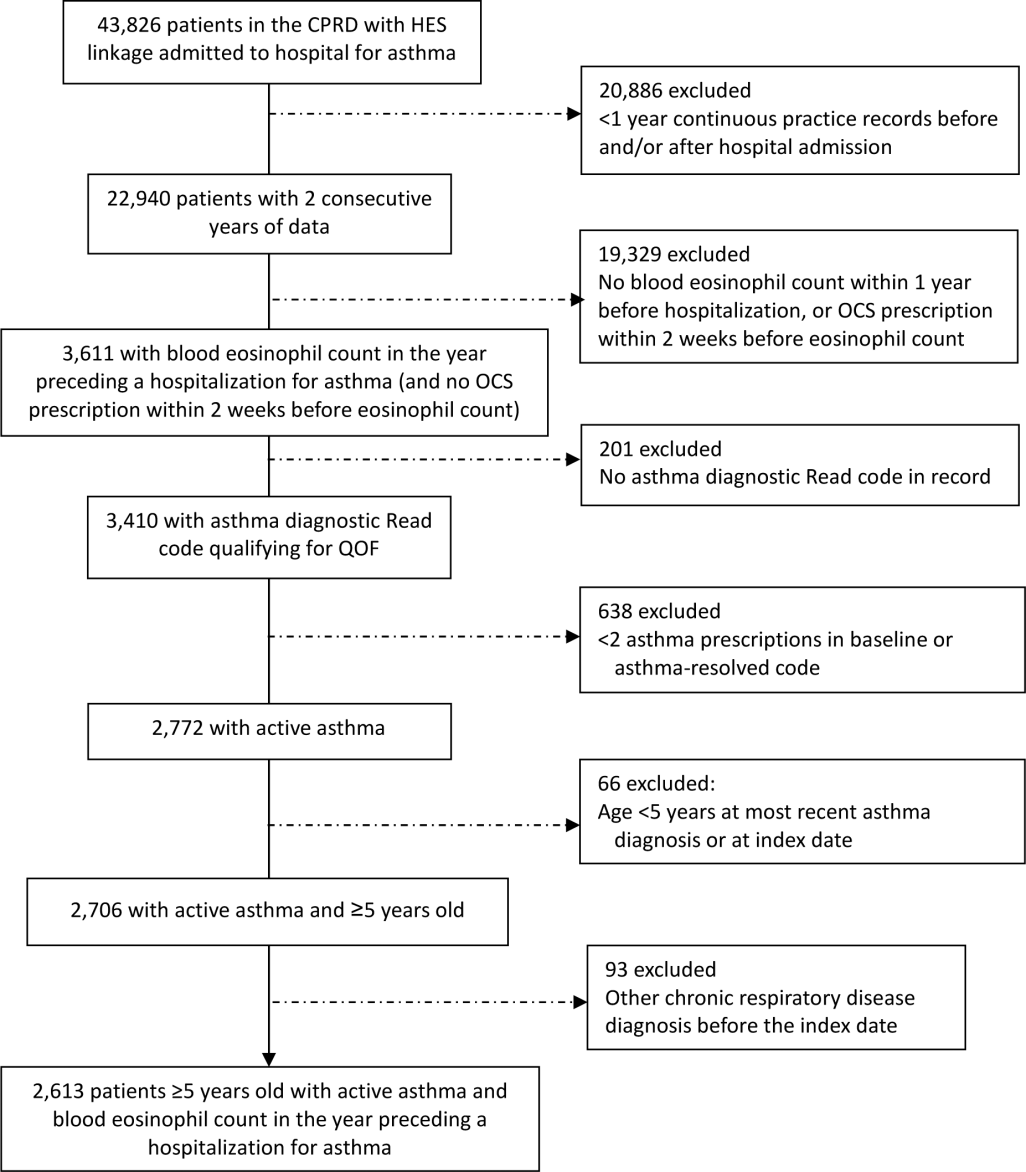
Flow diagram showing selection of eligible patients from the database. CPRD = Clinical Practice Research Database. HES = Hospital Episode Statistics. OCS = oral corticosteroid. QOF = Quality Outcomes Framework.

Of 146,485 patients in the CPRD with HES data linkage, 22,940 (16%) patients had at least one hospital admission for asthma and ≥2 years of medical record data, and 3,611 patients (16%) of those hospitalized had an eosinophil count recorded within 1 year before the hospitalization (and no oral corticosteroid prescription within 2 weeks prior). Of these 3,611 patients, 2,613 patients (72%) were ≥5 years old, had active asthma, and were eligible for the study (Fig 2).

In the study population, 482 of 2,613 patients (18%) were discharged from hospital on the same day. Six patients died (one patient died 31 weeks after readmission for asthma and was not censored; others were censored) during 1 year of follow up.

Characteristics of the total population with blood eosinophil count (n = 2,613) and 13,016 patients with asthma who met all eligibility criteria except availability of blood eosinophil count during baseline are presented in S2 Table. There were multiple statistically significant differences between the two groups of patients. Eligible patients with recorded eosinophil count were older than the 13,016 patients without eosinophil count (median age, 50 vs. 33 years), more commonly female (1,997/2,613, 76% vs. 7,542/13,016, 58%), heavier (mean BMI 29.1 vs. 26.0 kg/m^2^), and receiving a higher median ICS dose (219 vs. 132 μg/day, fluticasone-propionate equivalent) during the baseline year (S2 Table).

A high blood eosinophil count (≥0.35x10^9^ cells/L) was recorded during the year before the hospital admission for 835 of 2,613 patients (32%). The high blood eosinophil cohort had a median age of 45 (vs. 54 years in the cohort with eosinophil count of <0.35x10^9^ cells/L) and included proportionately fewer women and fewer overweight and obese patients (Table 1). In addition, patients with eosinophil count ≥0.35x10^9^ cells/L were more likely to be never-smokers and to have a recorded diagnosis of rhinitis, atopic eczema, or nasal polyps.

**Table 1:** Baseline demographic and clinical characteristics.

	All patients (N = 2,613)	Blood eosinophil cohort		
Variable		<0.35x10^9^ cells/L (n = 1,778)	≥0.35x10^9^ cells/L (n = 835)	P value^a^
Age				
Median (IQR)	51.0 (36.0–69.0)	54.0 (39.0–70.3)	45.0 (30.0–65.0)	<0.0001
5–12 years	56 (2.1)	17 (1.0)	39 (4.7)	<0.0001
13–17 years	77 (2.9)	31 (1.7)	46 (5.5)	
18–64 years	1,681 (64.3)	1,141 (64.2)	540 (64.7)	
≥65 years	799 (30.6)	589 (33.1)	210 (25.1)	
Female sex	1,997 (75.7)	1,392 (78.3)	585 (70.1)	<0.0001
Smoking status^b^				
Data available	2,597 (99.4)	1,771 (99.6)	826 (98.9)	
Current smoker	547 (21.1)	378 (21.3)	169 (20.5)	0.007
Ex-smoker	754 (29.0)	544 (30.7)	210 (25.4)	
Never smoker	1,296 (49.9)	849 (47.9)	447 (54.1)	
Body mass index^b^				
Data available	2,260 (86.5)	1,551 (87.2)	709 (84.9)	
Mean (SD)	29.2 (7.0)	29.6 (7.0)	28.4 (7.0)	<0.0001
<18.5 kg/m^2^	78 (3.5)	38 (2.5)	40 (5.6)	<0.0001
≥18.5 kg/m^2^ to <25 kg/m^2^	625 (27.7)	393 (25.3)	232 (32.7)	
≥25 kg/m^2^ to <30 kg/m^2^	625 (27.7)	450 (29.0)	175 (24.7)	
≥30 kg/m^2^	932 (41.2)	670 (43.2)	262 (37.0)	
Allergic/non-allergic rhinitis^c^	876 (33.5)	545 (30.7)	331 (39.6)	<0.0001
Atopic eczema^c^	927 (35.5)	595 (33.5)	332 (39.8)	<0.0001
Nasal polyps^c^	83 (3.2)	39 (2.2)	44 (5.3)	<0.0001
Chronic rhinosinusitis^c^	579 (22.2)	400 (22.5)	179 (21.4)	0.54
COPD^c^	284 (10.9)	192 (10.8)	92 (11.0)	0.87
GERD^c^	474 (18.1)	355 (20.0)	119 (14.3)	<0.001
Cardiovascular disease^c^	654 (25.0)	491 (27.6)	163 (19.5)	<0.0001
Charlson comorbidity index				
0	611 (23.4)	429 (24.1)	182 (21.8)	0.028
1–4	1,661 (63.6)	1,101 (61.9)	560 (67.1)	
≥5	341 (13.1)	248 (13.9)	93 (11.1)	
GINA step of asthma treatment^b^				
1	124 (4.7)	78 (4.4)	46 (5.5)	0.009
2	493 (18.9)	357 (20.1)	136 (16.3)	
3	468 (17.9)	298 (16.8)	170 (20.4)	
4	1,220 (46.7)	848 (47.7)	372 (44.6)	
5	308 (11.8)	197 (11.1)	111 (13.3)	
≥1 ICS inhaler prescribed	2,444 (93.5)	1,671 (94.0)	773 (92.6)	0.173
Daily dose of ICS (μg/day), median (IQR)^d^	262 (110–521)	263 (110–534)	247 (99–492)	0.041
≥1 SABA inhaler prescribed	2,432 (93.1)	1,646 (92.6)	786 (94.1)	0.144
Daily SABA dose, median (IQR)^d^	1.64 (0.82–3.55)	1.64 (0.66–3.29)	2.04 (0.82–4.11)	<0.0001
OCS daily dose (g), median (IQR)	0.55 (0–1.64)	0.55 (0–1.56)	0.55 (0–1.75)	0.139
No. severe asthma exacerbations				
0	747 (28.6)	516 (29.0)	231 (27.7)	0.25
1	848 (32.5)	589 (33.1)	259 (31.0)	
2	506 (19.4)	345 (19.4)	161 (19.3)	
3	266 (10.2)	174 (9.8)	92 (11.0)	
≥4	246 (9.4)	154 (8.7)	92 (11.0)	

Data expressed as No. (%) unless otherwise noted. COPD = chronic obstructive pulmonary disease. GERD = gastroesophageal reflux disease. GINA = Global Initiative for Asthma; ICS = inhaled corticosteroid; OCS = oral corticosteroid; SABA = short-acting β-agonist.

^a^P-value comparing blood eosinophil cohorts, computed from χ^2^ test for categorical variables, or Mann-Whitney test, for continuous variables. Where variables are presented as both continuous and categorical, the p-value is from the Mann-Whitney test.

^b^The closest BMI within 10 years of hospital discharge, and the smoking status closest to and within 5 years before hospital discharge, were included. The GINA treatment step was determined based on the last prescription before the hospitalization (S1 Table). The BMI categories applied to patients ≥18 years old; for children, BMI was not calculated because accurate information on age in months required to calculate BMI z-scores was not provided for privacy reasons.

^c^Comorbidities were those with diagnostic Read code ever-recorded in the available data before hospital discharge.

^d^ICS dose expressed as fluticasone propionate equivalent (μg/day), and one SABA dose defined as 200 μg in albuterol equivalents.

The likelihood of a blood eosinophil count being recorded was greater at dates closer to the hospital admission (S1 Fig). Patients with measurements within 4 weeks before the hospitalization were more likely to have a high blood eosinophil count (128/339, 38%) than those with measurement within a longer time period before the hospitalization (707/2274, 31%; p = 0.014). The length of time between recorded eosinophil count and admission with asthma as the primary diagnosis was greater in patients with high blood eosinophil counts than in patients without high counts, but the difference in distribution was not statistically significant (144 days [IQR, 56−250] vs. 131 days [58−229], p = 0.159).

The median duration of hospitalization (2 nights) was the same in patients with and without a high blood eosinophil count; however, there were fewer patients with a high blood eosinophil count who had a long hospital stay (Table 2).

**Table 2:** Duration of hospitalization.

	All patients (N = 2,613)	Blood eosinophil cohort		
Variable		<0.35x10^9^ cells/L (n = 1,778)	≥0.35x10^9^ cells/L (n = 835)	P value^a^
Nights in hospital, median (IQR)		2 (1–5)	2 (1–4)	
No. nights in hospital, n (%)				
0	482 (18.4)	323 (18.2)	159 (19.0)	0.006
1	529 (20.2)	349 (19.6)	180 (21.6)	
2	356 (13.6)	230 (12.9)	126 (15.1)	
3	281 (10.8)	182 (10.2)	99 (11.9)	
4	243 (9.3)	162 (9.1)	81 (9.7)	
5	149 (5.7)	99 (5.6)	50 (6.0)	
6	142 (5.4)	106 (6.0)	36 (4.3)	
≥7	431 (16.5)	327 (18.4)	104 (12.5)	

^a^P-value comparing blood eosinophil cohorts computed from χ^2^ test.

### Readmissions by eosinophil cohort

**Fig 3:**
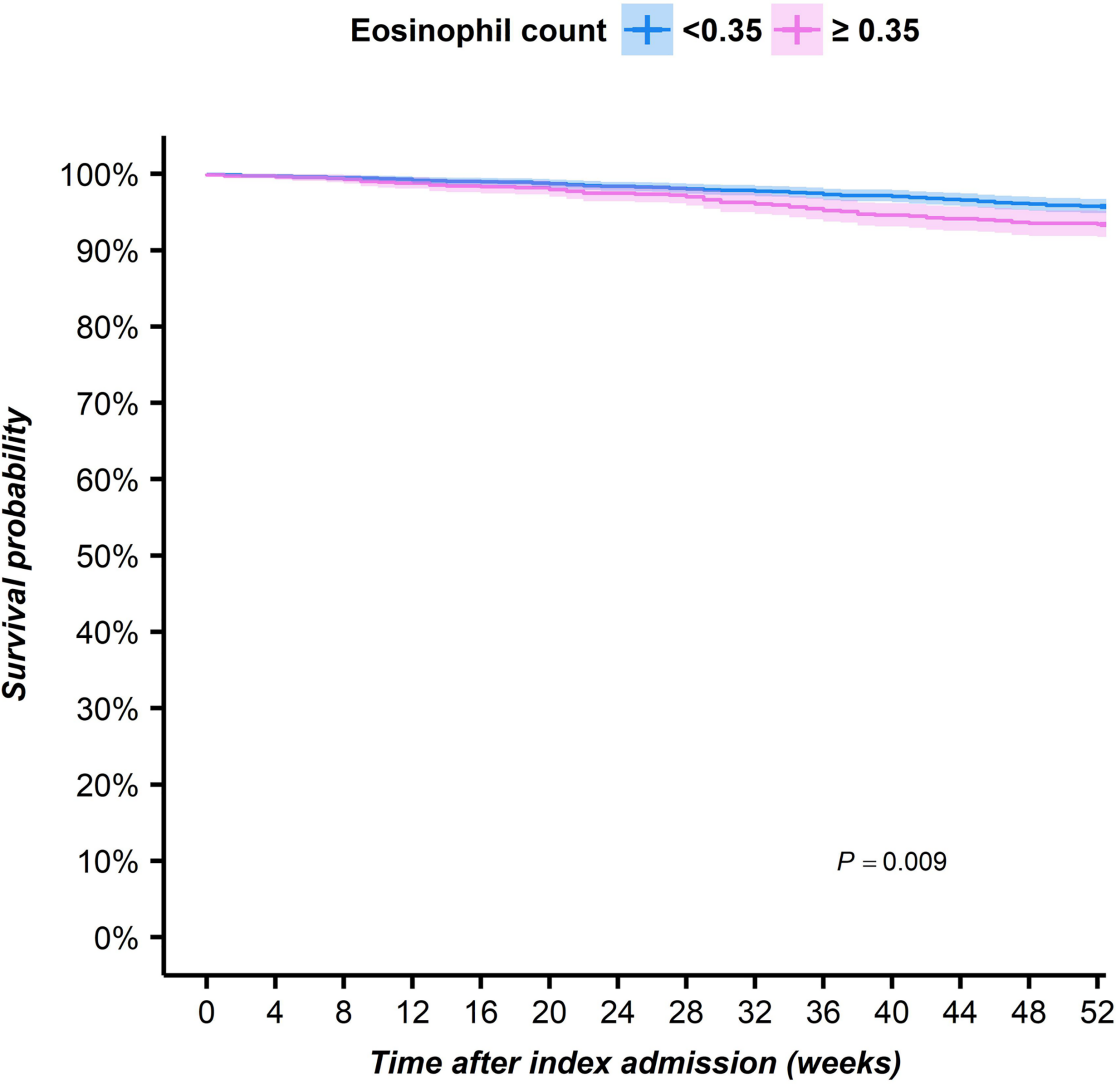
Kaplan-Meier curves describing the cumulative “survival” of a readmission to hospital for asthma in the first year after an admission with asthma as the primary diagnosis in patients with and without high blood eosinophil count.

Only 6 patients were readmitted to the hospital within 4 weeks of the first admission, with no significant difference between blood eosinophil cohorts (Table 3). At 1 year, 130 of 2,613 (5%) patients overall were readmitted for asthma, including a significantly greater percentage of patients with high vs. normal blood eosinophil count (Table 3; Fig 3). Patients with eosinophil count of ≥0.35x10^9^ cells/L had a 49% higher adjusted risk of readmission to hospital for asthma in the first year of follow-up than patients without a high count (HR 1.49; 95% CI 1.04–2.13; p = 0.029; Table 3).

**Table 3:** Readmissions to hospital within 4 weeks and 1 year and hazard ratios for readmission in the high eosinophil count cohort.

	Eosinophil cohort				
Readmission	<0.35x10^9^ cells/L (n = 1,778)	≥0.35x10^9^ cells/L (n = 835)	P value^a^	Adjusted HR (95% CI) for blood eosinophil count ≥0.35x10^9^/L^b^	P value
With asthma as primary diagnosis (n = 2,613)					
Within 4 weeks	4 (0.2)	2 (0.2)	0.94	—	—
Within 1 year	75 (4.2)	55 (6.6)	0.009	1.49 (1.04–2.13)	0.029
By known smoking status (n = 2,597)^c^					
Never-smokers (n = 1,296)	29 (3.4)	30 (6.7)	0.007	2.16 (1.27–3.68)	0.005
Ex-smokers (n = 754)	19 (3.5)	13 (6.2)	0.010	1.49 (0.73–3.06)	0.27
Current smokers (n = 547)	27 (7.1)	12 (7.1)	0.99	1.00 (0.49–2.04)	0.997
Never/ex-smokers pooled (n = 2,050)	48 (3.4)	43 (6.5)	0.002	1.78 (1.17–2.73)	0.007
With respiratory condition other than asthma, and asthma as subsidiary diagnosis (n = 2,613)					
Within 4 weeks	22 (1.2)	8 (1.0)	0.53	—	—
Within 1 year	81 (4.6)	39 (4.7)	0.90	1.12 (0.76–1.65)	0.57

^a^P-value computed using χ^2^ test.

^b^Adjusted for sex, age, smoking status, timing of blood eosinophil count measurement, duration of index hospitalization.

^c^16 patients with no recent record of smoking status were excluded from the analyses by smoking status.

### Interaction with smoking status

The effect of current smoking was non-significant (p = 0.073) when tested by including an interaction term for current smoking (yes/no) and high blood eosinophil count (yes/no) into the model. The increased readmission rate with a high blood eosinophil count was found only in non-smokers (HR 1.84; 1.20–2.80; p = 0.005) and not in current smokers (HR 0.88; 0.44–1.76; p = 0.73). In this analysis of all 2,613 patients, 16 patients without recent, recorded smoking status were included as non-smokers (never-smokers plus ex-smokers).

Results were similar for patients with known smoking status, with a significant 216% higher adjusted risk of readmission for never-smokers with high blood eosinophil count, and no additional risk for current smokers with high blood eosinophil count (Table 3). Although the association was most pronounced in never-smokers, no significant difference in the association was found between never-smokers and ex-smokers (p = 0.67) in the 2,050 patients recorded as not currently smoking.

### Sensitivity analyses

A high blood eosinophil count was recorded for 1,328 patients (51%) when defined as ≥0.25x10^9^ cells/L, and for 588 patients (23%) when defined as ≥0.45x10^9^ cells/L. The association between a high blood eosinophil count and readmission to hospital for asthma was less pronounced and not significant for patients with blood eosinophil count of either ≥0.25x10^9^ cells/L (HR = 1.17; 0.82−1.66; p = 0.39) or ≥0.45x10^9^ cells/L (HR = 1.15; 0.77−1.72; p = 0.50; S3 Table). The association was also not significant in never-smokers or in never/ex-smokers combined using either definition of high blood eosinophil count (S3 Table).

A total of 169 of the 2,613 patients (6%) had no prescription for ICS in the baseline year before being hospitalized for asthma; of the 169, 115 (68%) had ICS prescribed in the outcome year. After exclusion of these 115 patients, HRs for the association with blood eosinophil count of ≥0.35x10^9^ cells/L slightly increased as compared with those for the full population (S3 Table). The HR was 1.77 (95% CI, 1.15−2.72; p = 0.009) for never/ex-smokers combined, which was very similar to the HR for never/ex-smokers combined of the full population (1.78). However, effect modification by current smokers was not significant in this subpopulation (p = 0.28).

Results of an additional subanalysis excluding patients with a concomitant diagnosis of COPD showed no relevant difference in association for the remaining 2,329 patients (HR = 1.48; 95% CI 1.01–2.17, p = 0.045; see S3 Table).

## Discussion

In this large, historical cohort study, we found that patients who had a blood eosinophil count of ≥0.35x10^9^ cells/L recorded in the year preceding an asthma-related hospitalization had a significantly greater risk of readmission for asthma during the year after they were discharged. Few patients (n = 6) were readmitted to hospital for asthma within 4 weeks after discharge, while by 1 year after discharge, 5% (130 of 2,613) patients were readmitted for asthma. The greater risk of readmission during 1 year follow-up was present only for patients with high blood eosinophil count who were never- or ex-smokers (not for current smokers).

Our study is one of few studies examining hospital readmissions for asthma in a general asthma population and in the real-life setting. Readmissions in the present study were comparatively infrequent relative to results in other studies: for example, in one US study, approximately 4% of patients were readmitted for an asthma exacerbation within 30 days [21], and in France from 2002–2005, 15% were readmitted for asthma within 1 year [22]. The overall rate of hospital admissions for asthma in England appears to be lower that than for Western Europe as a whole, the latter reported in 2004 to be 7% [1,23].

Other recent studies of hospital readmissions have been limited to patients on systemic corticosteroids [9], have examined readmissions up to only 30 days [11,12,24], were much smaller [24], and/or were conducted at a single institution [25,26]. None of these studies, nor others examining readmissions after 30 days [27–29], examined the association of hospital readmissions with blood eosinophil count. While Gonzalez-Barcala et al. [13] in their retrospective study at a single hospital in Spain found differently from the present study that elevated eosinophil count was associated with a lower incidence of readmissions, it is difficult to compare their study with ours because of differences in methods. For example, the reference blood eosinophil count was that taken upon admission rather than before hospitalization during a baseline year, and the length of the follow-up period for analyzing readmissions is unclear [13].

An interesting finding in the present study that requires further investigation is the effect of smoking status on association of readmissions with eosinophil count. Cigarette smoking increases levels of oxidative stress, alters airway immune responses, and increases risk of hospitalization in patients with asthma [30]. Westerhof et al. [31] in their study of patients with severe asthma found that frequent exacerbations were associated with blood eosinophil count only in never smokers and not in ex-smokers, for whom blood neutrophil count was an independent predictor of frequent exacerbations (smokers not studied). In our study, both never- and ex-smokers (but not current smokers) who had a high eosinophil count were at greater risk of asthma-related readmission, although for ex-smokers separately this association was not statistically significant. Moreover, in our study the difference in association between non-smokers (never-plus ex-smokers pooled) and smokers was large and statistically significant. Clearly, additional work is needed to examine biomarker and peripheral blood cell profiles in relation to smoking status and hospital readmissions and other asthma-related outcomes.

The median duration of hospitalization (2 nights) was the same in both normal and high blood eosinophil cohorts; however, patients with a high blood eosinophil count were less likely to have a hospital stay longer than 5 nights (17% vs. 24% of those without high eosinophil count). This finding illustrates the conundrum of eosinophilic asthma: while it tends to be more severe in terms of exacerbations and asthma control, eosinophilic asthma is also potentially more responsive to therapies targeting type 2 inflammation, including ICS and biologics.

We speculated that the association between eosinophil count and readmission could be diluted for patients with eosinophil count performed several months before the first admission; therefore, we re-examined outcomes including only patients with eosinophil counts measured close to the initial hospitalization to see if the association were stronger. However, when selecting those with eosinophil count recorded within 4 months before hospitalization, the numbers became small and associations non-significant, although the direction of the effect was the same: for never- and ex-smokers pooled (n = 915), the risk of readmission was 51% greater but non-significant (adjusted HR 1.69;0.60–4.76; p = 0.32).

A strength of this study is that we included a broad patient population with asthma, not limited to those with severe asthma. We selected inclusion criteria to ensure that patients’ asthma was actively managed in advance of the hospital admission, thereby excluding patients experiencing a first episode of asthma diagnosed at the time of admission. Moreover, we required that patients had not received an oral corticosteroid prescription within 2 weeks before the eosinophil count to obviate the eosinopenic effects of systemic corticosteroids [32,33]. The data sources we used are well-regarded and frequently employed for pharmacoepidemiological studies [15–17,34]. The primary care data in the CPRD is considered to be high-quality, with recording that has been standardized and improved since the institution in 2004 of the UK Quality Outcomes Framework (QOF) [17], which provides financial incentives for GPs to deliver quality care, including an annual asthma review covering asthma control status, smoking, and inhaler technique. Detailed information about hospital admissions was drawn from HES, a data warehouse linked to the CPRD [16].

Nevertheless, a limitation is that the study dataset comprised information collected for clinical and routine use rather than specifically for research purposes. Moreover, prescriptions for drugs prescribed by specialists are not reliably recorded in the CPRD. Therefore, we could not evaluate treatment prescribed immediately after hospital discharge. However, the daily dose of ICS prescribed by GPs in the year after admission was not significantly different between patients with and without high eosinophil counts (median for both: 329 vs. 329 μg/day fluticasone-equivalent, p = 0.70, Mann-Whitney test). Finally, as for all observational studies, there is the possibility of residual confounding from unrecognized and/or unmeasured factors.

A “count-response” association of blood eosinophil levels with risk of asthma exacerbations has been reported in both an observational study [3] and for the placebo arm of clinical trials [35,36]. Our study had insufficient patient numbers to assess the presence of a count-response relationship with hospital readmissions using incremental categories to define high eosinophil count. Our definition of ≥0.35x10^9^ cells/L for high blood eosinophil count captured a clear association of high blood eosinophil count with risk of readmission, while there were fewer patients, hence limited statistical power, to evaluate the higher cut-point of ≥0.45x10^9^ cells/L, although the direction of the effect was the same. Alternatively, new ICS use or better ICS adherence after the index hospitalization might have reduced the effect of elevated eosinophil count; however, it would not be easy to quantify this possibility in the framework of a historical cohort study, and in spite of this possibility we found a strong association at the ≥0.35x10^9^ cells/L definition.

We did not exclude patients with a concomitant diagnosis of COPD; therefore, approximately one-tenth of the study population appeared to have some form of physician-diagnosed asthma-COPD overlap [37], although these patients were too few to analyze separately. However, the sensitivity analysis excluding these patients supported the findings for the full population.

By necessity we were able to include only patients who had a recorded blood eosinophil count, which is not routinely measured in clinical practice, a factor serving as a possible source of selection bias and thereby limiting the generalizability of our findings. There were large differences in baseline characteristics between the patients with available eosinophil count and those without, who tended to be younger; more likely female, a current smoker, and of normal weight; and less likely having comorbidities such as rhinitis, chronic sinusitis, gastroesophageal reflux disease, and cardiovascular disease. The age differences were expected because older people more frequently have full blood counts available. Further work is needed to examine the use of blood eosinophil count in the clinical assessment of the full spectrum of patients with asthma.

Tailoring asthma therapy using sputum eosinophil counts appears to be effective in reducing exacerbations, particularly for adults with frequent exacerbations [38]. Thus, blood eosinophil count, more practical to measure than sputum eosinophil count, could play a role in tailoring asthma therapy with the goal of reducing exacerbations, hence potentially hospital readmissions. Moreover, further research is needed to identify the mechanism(s) behind the increased risk of readmission associated with high blood eosinophil count, such as possible undertreatment with ICS or insufficient effectiveness of ICS. In addition, more specifically, a re-examination is needed of the absence of association with readmissions and high blood eosinophil count in current smokers, as there was limited statistical power in this subgroup of patients, reflected by the wide confidence interval.

## Conclusions

A high blood eosinophil count in the year before an asthma-related hospitalization is associated with increased risk of readmission within the following year. This risk was slightly greater in the subset of patients who were not new initiators of ICS treatment after their index hospital admission, suggesting that this trait is only partially treatable with anti-inflammatory therapy. This association was present only in non-smoking patients with high blood eosinophil count. Our findings support the benefit of including a full blood count with differential as a routine assessment in clinical practice for patients with not well-controlled asthma. Moreover, our findings support the need for careful follow-up, with treatment optimization, after hospital discharge for patients with asthma and preadmission high blood eosinophil count.

## Supporting information

S1 Fig Distribution of the number of days before hospital discharge on which the most recent eosinophil measurement was recorded. (TIF)

S1 Table Definitions applied for Global Initiative for Asthma (GINA) treatment step, determined using each patient’s last prescription(s) before the first hospital admission. (DOCX)

S2 Table Demographic and clinical characteristics of all eligible patients with blood eosinophil count and of patients meeting all eligibility criteria except availability of eosinophil count^a^. (DOCX)

S3 Table Readmissions for asthma within 1 year and hazard ratios for readmission in the high eosinophil count cohort: Sensitivity analyses. (DOCX)
