# Supplementary figures and images for: Association between blood eosinophil count and risk of readmission for patients with asthma: Historical cohort study
Source: PLoS One. 2018 Jul 25;13(7):e0201143. doi: 10.1371/journal.pone.0201143 (PMC6059485; doi:10.1371/journal.pone.0201143)

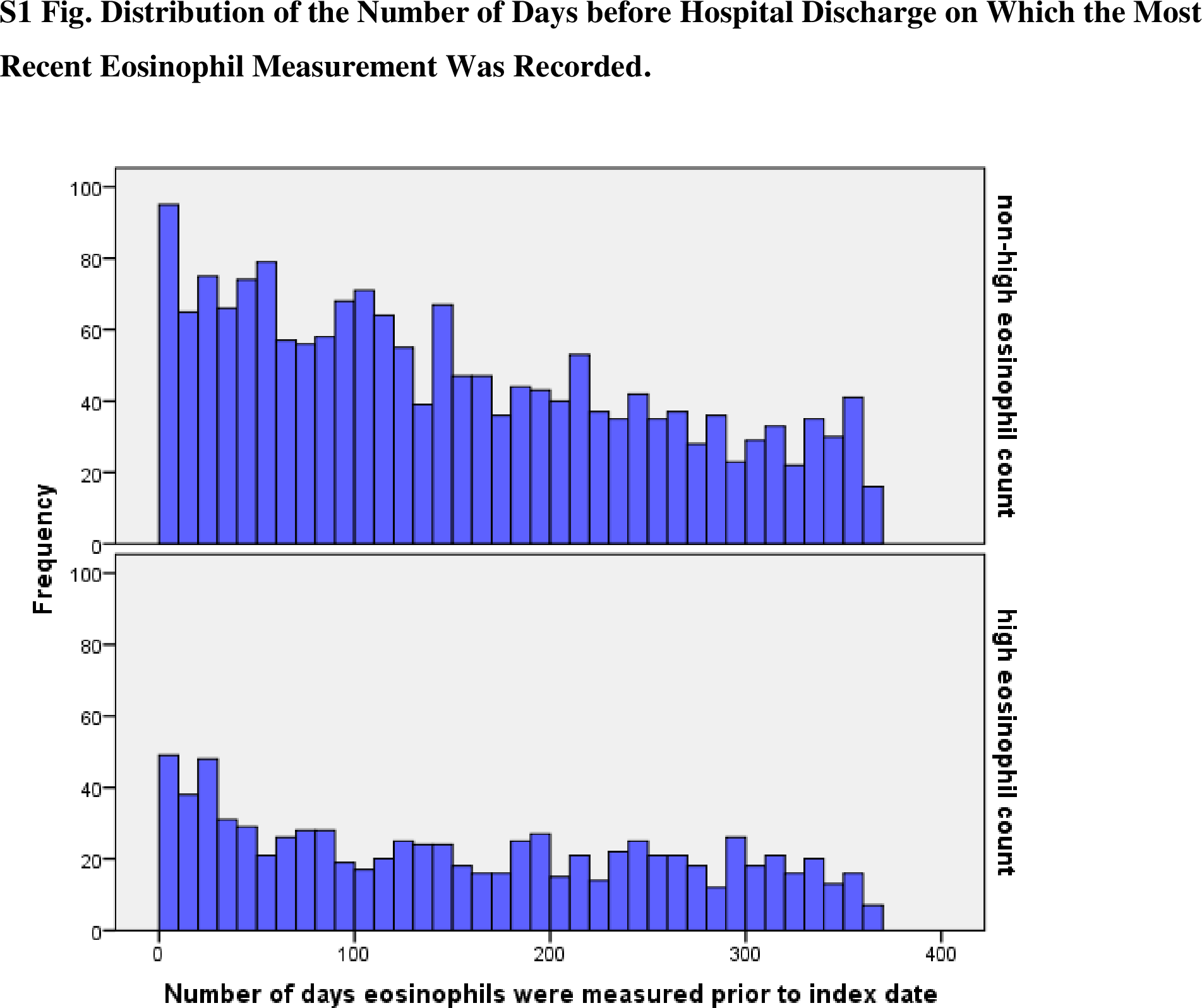

Supplement: S1 Fig — (TIF) [file pone.0201143.s001.tif]
