# Supplementary material for: Association between blood eosinophil count and risk of readmission for patients with asthma: Historical cohort study
Source: PLoS One. 2018 Jul 25;13(7):e0201143. doi: 10.1371/journal.pone.0201143 (PMC6059485; doi:10.1371/journal.pone.0201143)
Supplement: S1 Table — (DOCX) [file pone.0201143.s002.docx]

**S1 Table. Definitions Applied for Global Initiative for Asthma (GINA) Treatment Step, Determined Using Each Patient’s Last Prescription(s) Before the First Hospital Admission.**

| **GINA step** | **Treatment prescribed** |
| --- | --- |
| **Step 1** | Short-acting relievers only (β-agonist or muscarinic antagonist) |
| **Step 2** | Low-dose ICS^a^ without other controllers^b^ |
|  | LTRA without other controllers |
|  | Theophylline without other controllers |
|  | Sodium cromoglycate or nedocromil sodium |
| **Step 3** | Medium- or high-dose ICS without other controllers |
|  | Low-dose ICS / LABA |
|  | Low-dose ICS / LAMA |
|  | Low-dose ICS without LABA or LAMA plus [LTRA and / or theophylline] |
|  | LABA and / or LAMA without ICS |
|  | LTRA plus theophylline without ICS |
| **Step 4** | Medium- or high-dose ICS / LABA |
|  | Medium- or high-dose ICS / LAMA |
|  | Medium- or high-dose ICS plus [LTRA and / or theophylline] |
|  | Low-dose ICS / LABA plus ≥1 other controller |
|  | Low-dose ICS / LAMA plus ≥1 other controller |
|  | Three or more controllers without ICS |
| **Step 5** | Maintenance oral corticosteroids plus any other treatment |

ICS = inhaled corticosteroid.

^a^Low-, medium-, and high-dose ICS defined according to GINA guidelines (Global Initiative for Asthma. GINA Report, Global Strategy for Asthma Management and Prevention. http://ginasthma.org/. Accessed March 18, 2018.).

^b^Controllers included long-acting β-agonist (LABA), long-acting muscarinic antagonist (LAMA), leukotriene antagonist (LTRA), and theophylline.
