# Supplementary material for: Association between blood eosinophil count and risk of readmission for patients with asthma: Historical cohort study
Source: PLoS One. 2018 Jul 25;13(7):e0201143. doi: 10.1371/journal.pone.0201143 (PMC6059485; doi:10.1371/journal.pone.0201143)
Supplement: S2 Table — (DOCX) [file pone.0201143.s003.docx]

**S2 Table. Demographic and Clinical Characteristics of All Eligible Patients with Blood Eosinophil Count and of Patients Meeting All Eligibility Criteria Except Availability of Eosinophil Count.^a^**

| **Baseline variable** | **Eligible patients**  **(N = 2,613)** | **Hospitalized patients with no eosinophil count**  **(N = 13,016)** | **P*-*value** |
| --- | --- | --- | --- |
| Age |  |  |  |
| Median (IQR) | 50.0 (35.0-68.0) | 33.0 (14.0-54.0) | <0.0001 |
| 5–12 years | 87 (3.3) | 3,000 (23.0) | <0.0001 |
| 13–17 years | 86 (3.3) | 850 (6.5) |  |
| 18–64 years | 1,668 (63.8) | 7,122 (54.7) |  |
| ≥65 years | 772 (29.5) | 2,044 (15.7) |  |
| Female sex | 1,977 (75.7) | 7,542 (57.9) | <0.0001 |
| Smoking status^b^ |  |  |  |
| Data available | 2,597 (99.4) | 12,255 (94.2) | <0.0001 |
| Current smoker | 1,258 (48.4) | 5,882 (48.0) |  |
| Ex-smoker | 613 (23.6) | 3,589 (29.3) |  |
| Never smoker | 726 (28.0) | 2,784 (22.7) |  |
| Body mass index^b^ |  |  |  |
| Data available | 2,240 (85.7) | 8,756 (67.3) |  |
| Mean (SD) | 29.1 (7.0) | 26.0 (7.1) | <0.0001 |
| <18.5 kg/m^2^ | 86 (3.8) | 1,260 (14.4) | <0.0001 |
| ≥18.5 kg/m^2^ to <25 kg/m^2^ | 622 (27.8) | 3,018 (34.5) |  |
| ≥25 kg/m^2^ to <30 kg/m^2^ | 628 (28.0) | 2,214 (25.3) |  |
| ≥30 kg/m^2^ | 904 (40.4) | 2,264 (25.9) |  |
| Allergic/non-allergic rhinitis^c^ | 810 (31.0) | 3,251 (25.0) | <0.0001 |
| Atopic eczema^c^ | 878 (33.6) | 4,307 (33.1) | 0.75 |
| Nasal polyps^c^ | 83 (3.2) | 461 (1.9) |  |
| Chronic rhinosinusitis^c^ | 75 (2.9) | 261 (2.0) | 0.0054 |
| COPD^c^ | 544 (20.8) | 1,408 (10.8) | <0.0001 |
| GERD^c^ | 255 (9.8) | 1,167 (9.0) | 0.20 |
| Cardiovascular disease^c^ | 425 (16.3) | 943 (7.2) | <0.0001 |
| Charlson comorbidity index |  |  |  |
| 0 | 609 (23.3) | 3,218 (24.7) | <0.0001 |
| 1–4 | 1,694 (64.8) | 9,272 (71.2) |  |
| ≥5 | 310 (11.9) | 526 (4.0) |  |
| GINA step of asthma treatment^b^ |  |  |  |
| 1 | 139 (5.3) | 1,097 (8.4) | <0.0001 |
| 2 | 563 (21.5) | 3,464 (26.6) |  |
| 3 | 565 (21.6) | 3,658 (28.1) |  |
| 4 | 1,107 (42.4) | 3,914 (30.1) |  |
| 5 | 239 (9.1) | 883 (6.8) |  |
| ≥1 ICS inhaler prescribed | 2,417 (92.5) | 11,557 (88.8) | <0.0001 |
| Daily dose of ICS (µg/day), median (IQR)^d^ | 219 (88–491) | 132 (55–329) | <0.0001 |
| ≥1 SABA inhaler prescribed | 2,451 (93.8) | 12,465 (95.8) | <0.0001 |
| Daily SABA dose, median (IQR)^d^ | 1.6 (0.8–3.3) | 1.6 (0.7–3.3) | 0.75 |
| OCS daily dose (g), median (IQR) | 0.4 (0.0–1.4) | 0.2 (0.0–1.0) | <0.0001 |
| Number of severe asthma exacerbations |  |  |  |
| 0 | 792 (30.3) | 5,211 (40.0) | <0.0001 |
| 1 | 873 (33.4) | 4,138 (31.8) |  |
| 2 | 499 (19.1) | 1,974 (15.2) |  |
| 3 | 250 (9.6) | 913 (7.0) |  |
| ≥4 | 199 (7.6) | 780 (6.0) |  |

Data expressed as No. (%) unless otherwise noted. COPD = chronic obstructive pulmonary disease; GERD = gastroesophageal reflux disease; GINA = Global Initiative for Asthma; ICS = inhaled corticosteroid; OCS = oral corticosteroid; SABA = short-acting β-agonist.

^a^For these comparisons, we assessed the first hospital admission in the database for each patient, also for the 2,613 patients included in the study, regardless of availability of eosinophil counts in the baseline year. For 2,076 of these 2,613 patients (79%) this was the same admission as that analyzed and reported in the main paper. Instead, for 537 patients (21%), the baseline characteristics refer to those at their first recorded hospitalization, while a later admission (when they had a blood eosinophil count recorded during the prior year) was used for the main analyses. Hence there are differences between baseline characteristics for eligible patients as reported in Table 1.

^b^The closest BMI within 10 years of hospital discharge, and the smoking status closest to and within 5 years before hospital discharge, were included. The GINA treatment step was determined based on the last prescription before the hospitalization (see Table S1). The BMI categories applied to patients ≥18 years old. For children BMI was not calculated because accurate information on age in months required to calculate BMI z-scores was not provided for privacy reasons.

^c^Comorbidities were those with diagnostic Read code ever-recorded in the available data before hospital discharge.

^d^ICS dose expressed as fluticasone propionate equivalent (µg/day), and one SABA dose defined as 200 µg in albuterol equivalents.
