# Supplementary material for: Association between blood eosinophil count and risk of readmission for patients with asthma: Historical cohort study
Source: PLoS One. 2018 Jul 25;13(7):e0201143. doi: 10.1371/journal.pone.0201143 (PMC6059485; doi:10.1371/journal.pone.0201143)
Supplement: S3 Table — (DOCX) [file pone.0201143.s004.docx]

**S3 Table. Readmissions for Asthma within 1 Year and Hazard Ratios for Readmission in the High Eosinophil Count Cohort: Sensitivity Analyses.**

| **Readmission** | **Adjusted HR**  **(95% CI)** | **P value** |
| --- | --- | --- |
| Applying different definitions of high blood eosinophil count (n=2,613) |  |  |
| High eosinophil count defined as ≥0.25x10^9^ cells/L | 1.17 (0.82−1.66)^a^ | 0.39 |
| By known smoking status: |  |  |
| Never/ex-smokers pooled (n=2,050) | 1.31 (0.86−2.00) | 0.21 |
| Never-smokers (n=1,296) | 1.57 (0.92−2.69) | 0.10 |
| High eosinophil count defined as ≥0.45x10^9^ cells/L | 1.15 (0.77−1.72)^b^ | 0.50 |
| By known smoking status: |  |  |
| Never/ex-smokers pooled (n=2,050) | 1.49 (0.93−2.37) | 0.096 |
| Never-smokers (n=1,296) | 1.71 (0.97−3.03) | 0.063 |
| Excluding 115 patients initiated on ICS after the first hospitalization (n=2,498) |  |  |
| High eosinophil count defined as ≥0.35x10^9^ cells/L (n=2,498) | 1.61 (1.12−2.32)^a^ | 0.008 |
| By known smoking status: |  |  |
| Never/ex-smokers pooled (n=1,964) | 1.77 (1.15−2.72) | 0.009 |
| Excluding 284 patients with prior COPD diagnosis (n=2,329) |  |  |
| High eosinophil count defined as ≥0.35x10^9^ cells/L (n=2,329) | 1.48 (1.01−2.17)^a^ | 0.045 |
| By known smoking status: |  |  |
| Never/ex-smokers pooled (n=2,079) | 1.52 (1.02−2.27) | 0.040 |

HR = hazard ratio; ICS = inhaled corticosteroid.

^a^Adjusted for sex, age, smoking status, timing of blood eosinophil count measurement, duration of index hospitalization.

^b^Adjusted for sex, age, smoking status, body mass index, timing of blood eosinophil count measurement, duration of index hospitalization.
